# A Meta-Analysis on Sex Differences in Resting-State Vagal Activity in Children and Adolescents

**DOI:** 10.3389/fphys.2017.00582

**Published:** 2017-08-24

**Authors:** Julian Koenig, Joshua A. Rash, Tavis S. Campbell, Julian F. Thayer, Michael Kaess

**Affiliations:** ^1^Section for Translational Psychobiology in Child and Adolescent Psychiatry, Department for Child and Adolescent Psychiatry, Centre of Psychosocial Medicine, University of Heidelberg Heidelberg, Germany; ^2^Department of Psychology, University of Calgary Calgary, AB, Canada; ^3^Department of Psychology, Memorial University of Newfoundland St. John's, NL, Canada; ^4^Department of Psychology, The Ohio State University Columbus, OH, United States; ^5^University Hospital of Child and Adolescent Psychiatry and Psychotherapy, University of Bern Bern, Switzerland

**Keywords:** vagal activity, heart rate variability, sex differences, children, adolescents

## Abstract

Lower vagal activity is associated with psychopathology independent of age. Research suggests that alterations of vagal activity precede the development of psychopathology. The present review aimed to quantify sex differences in vagal activity in children and adolescents. Studies reporting on sex differences on measures of vagally-mediated heart rate variability derived from short-term recordings under resting conditions in boys and girls were included. Drawing on data from more than 5,000 children and adolescents, we provide evidence that healthy young girls display lower vagal activity and greater mean heart rate compared to boys, a finding that may have implications for risk associated with the development of internalizing psychopathology and somatic ill-health.

## Introduction

There are marked sex differences in the prevalence of adolescent psychiatric disorders. For example, mood, anxiety, and eating disorders usually have an onset during adolescence, and are more prevalent among girls (Zahn-Waxler et al., 2008; Merikangas et al., 2009).

Reduced resting state vagal activity, indexed by measures of high frequency heart rate variability [HF-HRV; rapid variability in heart rate (HR) observed with spontaneous respiration that is also referred to as respiratory sinus arrhythmia; RSA], has been linked to a variety of mental health conditions in adults (Malik and Camm, 2007). For example, reduced HF-HRV compared to healthy controls has been reported in depression (Kemp et al., 2010), anxiety disorders (Chalmers et al., 2014), and Borderline Personality Disorder (BPD) (Koenig et al., 2016b). Among children and adolescents, lower resting HF-HRV is observed in autism spectrum disorder (Neuhaus et al., 2014), conduct disorder (Beauchaine et al., 2001, 2007), BPD (Koenig et al., 2017b), and depression (Koenig et al., 2016a) but not attention deficit hyperactivity disorder (Koenig et al., 2017a). A study of autonomic function in a population cohort reported that HF-HRV was higher in adolescents with externalizing problems relative to adolescents with internalizing problems (Dietrich et al., 2007). Resting state vagal activity, indexed by HF-HRV, has been shown to be related to individual differences in the perception of emotional stimuli (Park et al., 2013; Park and Thayer, 2014) and to predict affective instability in daily life (Koval et al., 2013). As several studies have shown, reduced HF-HRV is associated with difficulties in emotion regulation among child, adolescent, and adult samples (see e.g., Berna et al., 2014; Beauchaine, 2015; Williams et al., 2015). Thus, HF-HRV may have a particular association to the internalizing disorders associated with difficulties in emotion regulation. With respect to temporal sequencing, research from the Whitehall II longitudinal study suggests that altered vagal activity, as indexed by HF-HRV, may precede the development of internalizing psychopathology such as depression (Jandackova et al., 2016). Similar, it has recently been shown, that reduced HF-HRV significantly predicted increased depressive symptoms across 1 year in a sample of 73 adolescents (Vazquez et al., 2016). Beyond psychiatric disorders, decreased resting state HRV is associated with a poorly functioning anti-inflammatory reflex (Pavlov and Tracey, 2012), increasing risk for physical ill-health in general (see Kemp and Quintana, 2013 for a review) and cardiovascular disease (CVD) in particular, the latter being the leading cause of death and disability worldwide (Thayer et al., 2010).

Given that parasympathetic and sympathetic influence on the heart operate by different signaling transmitters (i.e., acetylcholine and norepinephrine, respectively) with different latencies of onset, only parasympathetic influences can account for rapid changes in HR and its variability that are often observed during spontaneous respiration (Berntson et al., 1997). As such, power in the HF-HRV band and time-domain measures reflecting fast changes in the inter-beat interval (IBI) series (i.e., the root mean square of successive differences, RMSSD) are regarded as indices of vagal activity under resting conditions. In support of this, HF-HRV is nearly abolished by cholinergic blockade and functional vagotomy, confirming that HF-HRV is mediated primarily by change in vagal cardiac nerve traffic (Pomeranz et al., 1985; Berntson et al., 1997). While the interpretation of activity in the low-frequency (LF) band remains equivocal (Goldstein et al., 2011; Rahman et al., 2011), it is often considered to reflect both, activity of the sympathetic and parasympathetic branches of the autonomic nervous system (ANS).

Recently, we demonstrated substantial sex differences in time- and frequency-domain measures of HRV in healthy human adults using a meta-analytical approach drawing on data from a cumulative total of 296,247 subjects (Koenig and Thayer, 2016). Relative to men, the autonomic control of the women's heart is characterized by significantly less total power in the power spectral density (PSD) containing greater HF- and less LF-power. This was further reflected in a lower LF/HF ratio. Despite greater mean HR, women show higher levels of vagal parasympathetic activity relative to men. Most interestingly, sex differences emerged as a function of age and were more pronounced in older samples compared to younger samples. These results have important implications for research into the development of psychiatric disorders. Given that the prevalence of mood and anxiety disorders is higher in girls while the prevalence of externalizing disorders and substance abuse is higher in boys (Seedat et al., 2009), one would expect girls to show relatively lower parasympathetic vagal activity. Contributing to the *Frontiers Research Topic* addressing “*Heart Rate Variability and other Autonomic Markers in Children and Adolescents*,” the aim of the present review and meta-analysis was therefore to rereview the existing evidence on sex-differences in vagal activity, indexed by measures of vagally-mediated HRV, in children and adolescents under the age of 18. Sex differences in vagal activity at such young age may provide one mechanism contributing to differences in the prevalence of psychiatric disorders in children and adolescents.

## Methods

### Systematic search of the literature

The database of studies of a previous meta-analysis (Koenig and Thayer, 2016) was used for the present reanalysis. The initial systematic search of the literature was based on a review of four digital databases [PubMed, Web of Science (WOS), PsycINFO, and CINAHL Plus]. As indicated in the original report, the number of initial hits was recorded and after removing duplicates, abstracts of all identified articles were screened based on pre-defined inclusion criteria (Koenig and Thayer, 2016). Details on the literature search and criteria for inclusion are published elsewhere (Koenig and Thayer, 2016). For the present analysis, studies from the existing database were selected if the full-text reported (i) sufficient descriptive data (*see below*) of (ii) any given measure of vagally-mediated HRV, (iii) separately for boys and girls (sample mean age <18 years). Studies reporting overlapping samples were excluded. In case overlapping samples were reported by multiple titles, the earliest published report was included.

### Data extraction and meta-analysis

The name of the authors, the year of publication, sample size (total and by sex), and age were retrieved from all included studies. For the present meta-analysis we extracted all measures of vagally-mediated HRV reported, including time- (i.e., RMSSD) and frequency-domain (i.e., HF-HRV, RSA) measures. If studies reported measures of vagally-mediated HRV we also extracted data on mean HR and the mean IBI if reported. Descriptive data on the reported measures were extracted for female and male subjects separately. Measures of short-term recordings only were extracted. It is noted that guidelines for the measurement of HRV suggest “*analyses of short- and long-term electrocardiograms should always be strictly distinguished*” (Task Force of the European Society of Cardiology and the North American Society of Pacing Electrophysiology, 1996). For the present meta-analysis we focus on short-term recordings of resting state HF-HRV, as these are sought to reflect important trait influence (Bertsch et al., 2012) and are most frequently reported in the psychiatric literature.

Meta-analytical effect size estimates were based on means, standard deviations (*SD*), and the sample size (*n*). In case descriptive data was available other than as mean and SDs, data transformations were applied (Hozo et al., 2005; Wiebe et al., 2006; Higgins and Green, 2011). True effect estimates were computed as adjusted standardized mean differences (SMD, Hedge's g) for all measures. We undertook meta-analyses using *random-effect* models. Heterogeneity or inconsistency among trials in the magnitude or direction of effects estimated was investigated. Heterogeneity was assessed using the standard *I*^2^ *index, Chi*^2^, and *Tau*^2^ tests (Higgins and Thompson, 2002). Bias was further examined using funnel plots, illustrating the effect size (SMD) against standard error for asymmetry. Meta-analytic computations were performed using RevMan (*Version 5.3.4, Copenhagen: The Nordic Cochrane Centre, The Cochrane Collaboration, 2014*).

## Results

### Included studies

In historical order, Hedelin et al. (2000) assessed the effect of competitive cross-country skiing on HRV by testing HRV at rest and in response to tilt and exercise challenges before and after ski seasons among 17 adolescents. Grandjean et al. (2004) evaluated whether heart function in childhood is affected by exposure to methylmercury from seafood using a prospective cohort of 878 children assessed at 7 and 14 years of age. Cardiovascular function was assessed at both time points while children were in a relaxed supine condition. Gutin et al. (2005) assessed the effect of race, sex, physical activity, cardiovascular fitness, and adiposity on cardiac autonomic modulation in 304 healthy adolescents between 14 and 18 years of age. Wang et al. (2005) assessed whether HRV differed in Black and White children. Brunetto et al. (2005) evaluated the effects of aerobic fitness on HF-HRV in a sample of 41 adolescents aged 12–17 years. Participants were separated into tertiles based on aerobic fitness and HF-HRV was assessed at rest and in response to a maximal treadmill exercise test. The study by Greaves-Lord et al. (2007) evaluated the association between hyperarousal and symptoms of anxious and depressed mood using a nationally representative prospective cohort of Dutch children between 10 and 13 years of age. Reed et al. (2006) tested whether HRV in children differed by ethnicity (Caucasian and Asian) and sex. Sharshenova et al. (2006) recruited children between 9 and 10 years of age from three countries of different altitudes (1,650, 1,740, 2,030 m) to evaluate the effects of sex and altitude on HF-HRV.

Moodithaya and Avadhany (2012) evaluated sex differences in age-associated change in cardiac autonomic activity assessed via HRV using a cross sectional design. Michels et al. (2013) sought to provide age and sex specific reference values for short-term HRV data in children between 5 and 10 years of age. A secondary goal was to evaluate associations between HRV, physical fitness, BMI, and body composition. Tsao et al. (2013) assessed the effect of sex and age on conditioned pain modulation in a sample of 133 healthy children. Koch and Pollatos (2014) assessed the association between cardiac sensitivity and emotional intelligence in a sample of 1,350 children between 6 and 11 years of age. Jarrin et al. (2015) sought to derive normative HRV values stratified by age, sex, and HR for a population-based sample of children in Quebec. A summary of all included studies is provided in Table 1.

**Table 1:** Study and sample characteristics of studies reporting short-term heart rate variability; HF-HRV: high-frequency heart rate variability; RMSSD: root mean square of successive differences; NR: not reported; HR: heart rate; IBI: inter-beat-interval.

**Author and Year**	**Country**	**Sample size (Female)**	**Age**	**Inclusions**	**Exclusions**	**Other important factors**	**Adjustment for potential confounds**	**HRV recording**	**Recording duration**	**Tasks performed**	**Recording equipment**	**Processing equipment**	**HR/HRV metric**	**Breathing**	**HF-HRV frequency band**
Brunetto et al., 2005	Brasil	41 (21)	All: 15.3 (0.96);	Healthy	Smoker, Obesity, Hypertension, Medications, Diabetes, Syncope	Recruited from public schools.	No caffeine or alcohol on day of testing. No strenuous exercise on day prior to testing. Meal on day of testing was “light”	10-min resting baseline	5-min	Head tilt and incremental exercise task	Polar S810 (Polar Electro Oy, Finland)	Software developed by Yamamoto & Hughson	RMSSD; IBI	15 breaths per minute	0.15–0.4 Hz
Grandjean et al., 2004	Denmark	878 (440)	Sample assessed at 6.84 (0.31)– and 13.83 (0.32)–years	Sampled from a longitudinal cohort from the Faroe Islands	Neurological or other serious diseases; Low birth-weight.	NR	NR	Supine position	5-min	None	NEC-Sanei 1271SP ECG amplifier (Tokyo, Japan)	NR	HF-HRV; HR	Spontaneous respiration	0.15–0.4 Hz
Greaves-Lord et al., 2007	Netherlands	1027 (543)	All: 11(0.5)	10–13 years of age; Members of prospective “TRIALS” cohort	Severe mental retardation; Serious physical illness	NR	NR	Supine position	4-min	2-min standing	DAS-12 (Keithley Instruments Cleveland, OH)	CARSPAN Software	HF-HRV; HR	Spontaneous respiration	>0.14 Hz
Gutin et al., 2005	US	304 (171)	All: 16.24 (1.21);	Blacks and Whites; 14–18 years of age; Healthy	NR	NR	NR	Supine position with 10-min quiet rest	256 R-R intervals	None	Schiller electrocardiogrphic system (Schiller Ag, Baar, Switzerland)	NR	HF-HRV; RMSSD; HR	Spontaneous respiration	0.15–0.4 Hz
Jarrin et al., 2015	1036(555)	Canada	All: 10.2 (0.3);	Participants in a nationally representative Quebec longitudinal study	Medical pathology; born <24 or > 42 weeks gestation; Remote living	Representative random sampling was performed in Quebec	Physical activity, medication, caffeine intake within 24-h of testing	listed as “standardized protocol”	1-h	None	Marquette MARS 8500 Holter Monitor (Marquette Medical	Marquette Analysis workstation (Marquette Medical	HF-HRV; RMSSD; IBI; HR	NR	0.15–0.4 Hz
						Systems, Milwaukee, Wisconsin, USA)		Systems, Milwaukee, Wisconsin, USA)							
Hedelin et al., 2000	Sweden	17 (9)	NR	16–19 years of age; competitive cross-country skier	NR	Resting values were taken during controlled breathing	NR	Controlled breathing in supine position	5-min	Tilt table and exercise test	NR	Matlab (Mathworks Inc, Natick, MA)	HF-HRV; no HR/IBI (only during exercise)	12 breaths per minute	0.15–0.45 Hz
Koch and Pollatos, 2014	Germany	1350 (693)	All: 8.39 (0.94);	Members of longitudinal PIER study.	Missing data and technical problems (*n* = 305)	NR	NR	Resting	3-min	Heartbeat perception task	Polar RS800CX (Polar Electro Oy, Finland)	Polar ProTrainer 5	HF-HRV; RMSSD; HR	Spontaneous respiration	0.15–0.4 Hz
Michels et al., 2013	Belgium	460 (220)	All: 8.05 (NR)	Members of Belgin control region. 5-10 years of age;	Cardiovascular disease; Diabetes; Low quality HRV measurement	Physical activity; Normal breathing; Movement; Time of day	NR	Supine position	10-min	None	Polar Wearlink 31 (Polar Electro Oy, Finland)	Reported as “University of Kuopio Software”	HF-HRV; RMSSD; IBI; HR	Spontaneous respiration	0.15–0.4 Hz
Moodithaya and Avadhany, 2012	India	60 (30)	All: 9.4 (0.3);	Students and faculty at an institute in Bangalore	Hypertension; Diabetes; Chronic Disease; Oral contraceptive	Tested in morning after fasting; Refrain from smoking, caffeine, strenuous exercise (23-h)		Supine position	5-min	None	Biopac MP30 (Bipac Systems Inc, Santa Barbara, CA).	NR	HF-HRV; HR	Spontaneous respiration	0.15–0.4 Hz
Reed et al., 2006	62 (32)	Canada	All: 10.35 (0.6);	Participants drawn for a larger cohort enrolled in a school-based exercise program	CVD	NR	Tested in morning at school; No caffeine 2-h prior	Supine position	6-min (5 used)	None	Polar S810 (Polar Electro Oy, Finland)	Biomedicals Signal Analysis	HF-HRV; RMSSD; HR	NR	0.15–0.5 Hz
Sharshenova et al., 2006	113 (58)	listed as “3 countries”	All: 9 to 10 years of age	Healthy; Free of Chronic Illness	NR	Permanent residents from three countries tested at three different altitudes.		At rest	NR	during standing	NR	Reported as “specialy developed software”	HF-HRV; IBI	NR	0.15–0.4 Hz
Tsao et al., 2013	133 (70)	US	All: 13.0 (2.9);	Healthy children between 8 and 17 years of age	Medications; Chronic pain; Acute Illness; Developmental Delay	Data taken from a study of laboratory pain responses in children.	NR	Seated quietly watching neutral video	5-min	Laboratory pain tasks	Biopac System (Bipac Systems Inc, Santa Barbara, CA).	Kubios HRV Software	RMSSD; NO HR/IBI	NR	
Wang et al., 2005	385 (196)	US	All: 16.0 (2.0)	Members of Georgia Longitudinal Study or the George Cardiovascular Twin Study	Healthy; Free of Chronic Illness	Results are reported for White and Black participants	NR	Supine poition	256 R-R intervals	None	Schiller electrocardiogrphic system (Schiller Ag, Baar, Switzerland)	NR	HF-HRV; RMSSD; IBI	NR	0.15–0.4 Hz

### Excluded studies

Of the studies conducted in children/adolescents, five were excluded due to reporting on long-term recordings of HRV only (Han et al., 2000; Silvetti et al., 2001; Faulkner et al., 2003; Aziz et al., 2012; Rodríguez-Colón et al., 2014). Four studies had to be excluded for not reporting a measure of vagally-mediated HRV (Pikkujämsä et al., 1999; Lauritzen et al., 2008; Eyre et al., 2013; Faust et al., 2013). Further, one study reporting on fetuses was excluded (Kwon et al., 2014).

### Sex differences in vagal activity

**Figure 1:**
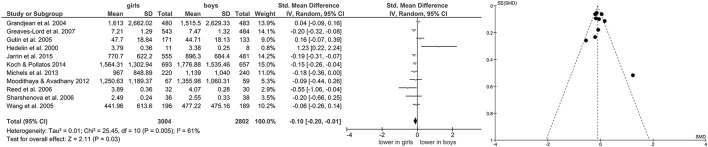
Forrest- and Funnel-Plot from Meta-Analysis on Short-Term Resting State HF-HRV; Grandjean et al. (2004): HF in ms^2^; data pooled for follow-up assessment (7 and 14 years of age), n of those with two assessments used before exclusion (unclear sex of excluded subjects); HF reported as geometric mean and interquartile range, transformed before pooling and analysis; Greaves-Lord et al. (2007): RSA in log ms^2^; supine data used for analysis; Gutin et al. (2005): HF in normalized units; data pooled across ethnic groups; Hedelin et al. (2000): HF in log ms^2^; supine data before training period (test 1) used; data reported as mean and range, transformed before analysis; Jarrin et al. (2015): HF in ms^2^; Koch and Pollatos (2014): HF in ms^2^; Michels et al. (2013): HF in ms^2^; data reported as mean and interquartile range, transformed before analysis; Moodithaya and Avadhany (2012): HF in ms^2^; data pooled for children and adolescents; data reported as mean and standard error of the mean, transformed before analysis; Reed et al. (2006): HF in log ms^2^; data pooled across ethnic groups; Sharshenova et al. (2006): HF in log ms^2^; supine data used for analysis; Wang et al. (2005): HF in ms^2^; data pooled across ethnic groups.

**Figure 2:**
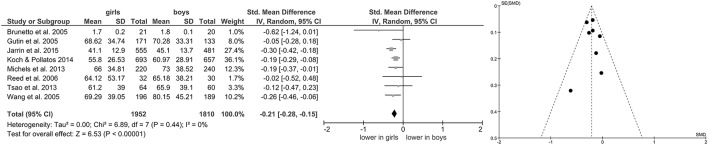
Forrest- and Funnel-Plot from Meta-Analysis on Short-Term Resting State RMSSD; Brunetto et al. (2005): supine data used for analysis; Gutin et al. (2005): data pooled across ethnic groups; Jarrin et al. (2015): data used as reported; Koch and Pollatos (2014): data used as reported; Michels et al. (2013): data reported as mean and interquartile range, transformed before analysis; Reed et al. (2006): data pooled across ethnic groups; Tsao et al. (2013): pre-task baseline data used; Wang et al. (2005): data pooled across ethnic groups.

Of studies screened for eligibility, 13 (Hedelin et al., 2000; Grandjean et al., 2004; Brunetto et al., 2005; Gutin et al., 2005; Wang et al., 2005; Reed et al., 2006; Sharshenova et al., 2006; Greaves-Lord et al., 2007; Moodithaya and Avadhany, 2012; Michels et al., 2013; Tsao et al., 2013; Koch and Pollatos, 2014; Jarrin et al., 2015) reported measures of vagally-mediated HRV derived from short-term recordings, yielding a total of 11 pooled comparisons for HF-HRV (Figure 1) and 8 comparisons for RMSSD (Figure 2). Meta-analysis showed a significant main effect for HF-HRV (Z = 2.11, *p* = 0.03; Hedges' g = −0.10, 95%CI [−0.20;−0.01], k = 11), comparing girls (*n* = 3,004) to boys (*n* = 2,802). Meta-analysis on RMSSD also yielded a significant main effect (Z = 6.53, *p* < 0.0001; Hedges' g = −0.21, 95%CI [−0.28;−0.15], k = 8), indicating lower resting state vagal activity in girls (*n* = 1,952) compared to boys (*n* = 1,810). While heterogeneity was present in the analysis of HF-HRV (Figure 1), respective tests indicated no heterogeneity for the analysis of RMSSD (Figure 2). Visual inspection of Funnel-Plots, revealed no significant publication bias for RMSSD but one outlier (Figure 1) in the analysis of HF-HRV (Hedelin et al., 2000). After removal of the outlier from analysis, meta-analysis on HF-HRV still showed a significant main effect (Z = 2.68, *p* = 0.007; Hedges' g = −0.11, 95%CI [−0.20;−0.03], k = 10; girls = 2,993; boys *n* = 2,794). Heterogeneity was moderate after removal of the outlier I^2^ = 52%.

### Sex differences in heart rate and mean inter-beat-interval

**Figure 3:**
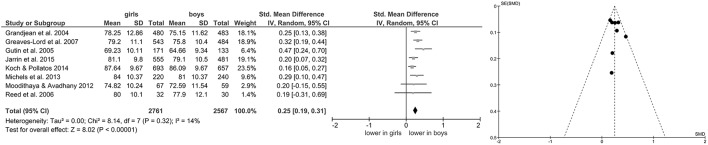
Forrest- and Funnel-Plot from Meta-Analysis on Short-Term Resting State HR; Grandjean et al. (2004): data pooled for follow-up assessment (7 and 14 years of age), n of those with two assessments used before exclusion (unclear sex of excluded subjects); Greaves-Lord et al. (2007): supine data used for analysis; Gutin et al. (2005): data pooled across ethnic groups; Jarrin et al. (2015): data used as reported; Koch and Pollatos (2014): data used as reported; Michels et al. (2013): data reported as mean and interquartile range, transformed before analysis; Moodithaya and Avadhany (2012): data pooled for children and adolescents; data reported as mean and standard error of the mean, transformed before analysis; Reed et al. (2006): data pooled across ethnic groups.

**Figure 4:**
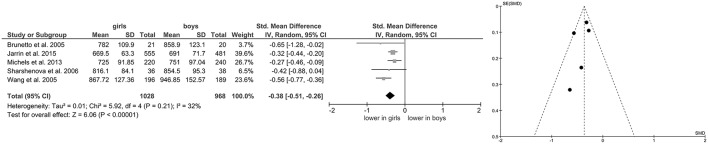
Forrest- and Funnel-Plot from Meta-Analysis on Short-Term Resting State IBI; Brunetto et al. (2005): supine data used for analysis; Jarrin et al. (2015): data used as reported; Michels et al. (2013): data reported as mean and interquartile range, transformed before analysis; Sharshenova et al. (2006): supine data used for analysis; Wang et al. (2005): data pooled across ethnic groups.

Of the included studies, eight also reported data on mean HR (Grandjean et al., 2004; Gutin et al., 2005; Reed et al., 2006; Greaves-Lord et al., 2007; Moodithaya and Avadhany, 2012; Michels et al., 2013; Koch and Pollatos, 2014; Jarrin et al., 2015) and five (Brunetto et al., 2005; Wang et al., 2005; Sharshenova et al., 2006; Michels et al., 2013; Jarrin et al., 2015) on mean IBI. Meta-analysis showed a significant main effect for mean HR (Z = 8.02, *p* < 0.0001; Hedges' g = 0.25, 95%CI [0.19;0.31], k = 8), indicating lower mean HR in boys (*n* = 2,567) compared to girls (*n* = 2,761), as illustrated in Figure 3. Meta-analysis on mean IBI also yielded a significant main effect (Z = 6.06, *p* < 0.0001; Hedges' g = −0.38, 95%CI [−0.51;−0.26], k = 5), indicating lower mean IBI in girls (*n* = 1,028) compared to boys (*n* = 968; Figure 4). No significant heterogeneity or publication bias was present for analysis on mean HR (Figure 3) and mean IBI (Figure 4).

## Discussion

### Main findings

We observed a small to moderate effect of sex on cardiac vagal activity derived from short-term, time-, and frequency-domain measures of HF-HRV among children and adolescents, such that girls displayed lower resting state cardiac vagal activity and greater mean HR relative to boys. Seven of the included studies initially reported significant sex differences on measures of vagal activity (Hedelin et al., 2000; Grandjean et al., 2004; Sharshenova et al., 2006; Moodithaya and Avadhany, 2012; Michels et al., 2013; Koch and Pollatos, 2014; Jarrin et al., 2015), with all but two studies (Hedelin et al., 2000; Grandjean et al., 2004) reporting higher vagally-mediated HRV in boys.

While the overall analysis of both HF-HRV and RMSSD suggest lower vagal activity in girls, the inconsistencies in the effect sizes reported between HF-HRV and RMSSD might be associated with measurement techniques. Frequency-domain measures of HRV provide information of different quality and detail compared to time-domain measures (Sinnreich et al., 1998). While RMSSD and HF-HRV are highly correlated (Goedhart et al., 2007), it has been suggested that time domain parameters can be estimated with less bias and variability then with frequency-domain parameters (Kuss et al., 2008). This issue relates, in part, to the influence of respiration on HF-HRV relative to RMSSD (Penttilä et al., 2001; Hill and Siebenbrock, 2009). The impact of respiration on HRV can be adjusted for during statistical modeling of short-term resting state HRV (Lewis et al., 2012), a procedure which is believed to yield a trait-like measure of vagal cardiac control that is relatively unaffected by situational constraints (Bertsch et al., 2012). Only two of the included studies on short-term recording controlled for respiration by instructing participants to breath in sequence with a metronome set at 15 breaths per minute (Brunetto et al., 2005) and 12 breaths per minute (Hedelin et al., 2000).

It is important to note that respiration rate decreases with increasing age (Fleming et al., 2011). In adults, the HF power band of the HRV power-spectrum is defined as the sum of the variance that occurs between 0.15 and 0.4 Hz. It is recommended that adjustment to these ranges be made for children and adolescents (Zisner and Beauchaine, 2017). All studies included in the present meta-analysis defined HF-HRV based on the frequency bands for adults (Hedelin et al., 2000; Grandjean et al., 2004; Gutin et al., 2005; Wang et al., 2005; Reed et al., 2006; Sharshenova et al., 2006; Greaves-Lord et al., 2007; Moodithaya and Avadhany, 2012; Michels et al., 2013; Koch and Pollatos, 2014; Jarrin et al., 2015). Given this lack of what is arguably most appropriate for HRV ranges (Fleming et al., 2011), future studies in children and adolescents should use time-domain measures of vagally-mediated HRV that are less affected by respiration, adjust frequency bands for spectral analysis or at least to record, and possibly control for respiration.

With respect to the focus of the Frontiers Research Topic, to “determined to what extent […] alterations of HRV during growth and development result from [changes in HR],” we like to emphasize differences in the association of HRV and HR by age. Albeit meta-analytical findings prohibit causal interpretations in the absence of raw longitudinal data, there are notable difference and similarities in autonomic cardiac control comparing findings in adults (Koenig and Thayer, 2016) and the present results. In our previous meta-analysis reporting on various HRV parameters across age groups, we observed evidence that HF-HRV (in normalized units and log-transformed) is greater in women relative to men, but observed no sex effects for RMSSD (Koenig and Thayer, 2016). In meta-regression, we observed that RMSSD was not affected by respiration, but sex differences reported for HF-HRV were attenuated among studies that did not adjust for respiration. Most interestingly, we observed that age was a significant covariate on RMSSD —while meta-analysis on RMSSD showed no consistent effects, adult women displayed greater RMSSD compared to men with increasing age. The present analysis extends these results by demonstrating that girls have lower RMSSD at younger ages when compared to boys. Two of the included studies directly addressed age dependent differences between girls and boys (Grandjean et al., 2004; Moodithaya and Avadhany, 2012), with equivocal conclusions. Both studies reported that LF, HF, and total power components of HRV decreased from childhood to adolescents (Grandjean et al., 2004) and further into adulthood (Moodithaya and Avadhany, 2012). In one study this decline was more pronounced for boys than girls (Grandjean et al., 2004). Importantly, only one of these four studies used a prospective design that allowed for the evaluation of the same children at each time-point (Grandjean et al., 2004) whereas the other studies were cross-sectional. Most interestingly, independent of age, we found greater mean HR in adult women (Koenig and Thayer, 2016) and girls compared to their male counterparts of the same age. Based on these findings, we carefully suggest that the association of HR and HRV, that in itself is the topic of ongoing scientific debate (Sacha and Pluta, 2008; Pradhapan et al., 2014; Sacha, 2014; Gąsior et al., 2015), is sensitive to age.

### Potential mechanisms

#### Pubertal development and sex hormones

There are many potential mechanisms underlying sex differences in resting state cardiac vagal activity in children and adolescents of which we will discuss two of the most prominent. While the pathophysiological mechanisms of altered vagal activity underlying psychopathology are not yet well-understood, the emerging influence of sex hormones during puberty may explain the observed differences between HF-HRV in boys and girls. A post-pubertal shift from lower vagal activity (pre-pubertal) to greater vagal activity (post-pubertal) in girls compared to boys may be related to hormonal changes during this sensitive period of development. The female sex hormones estrogen and progesterone are associated with cardiac autonomic modulation and animal models indicate that estrogen enhances cholinergic muscarinic activity and has a facilitating effect on cardiac vagal function (Du et al., 1994). Changes in estrogen levels in girls associated with pubertal development may explain the shift from lower vagal activity in girls (pre-pubertal) to relative higher vagal activity in women. Further, human research evaluating the influence of menstrual cycle on cardiac autonomic modulation in women who are not using oral contraceptives have indicated that variations in female sex hormones that occur across the menstrual cycle influence cardiac vagal activity (Hirshoren et al., 2002; Vallejo et al., 2005; Bai et al., 2009; Tenan et al., 2014). These studies suggest that the early follicular phase (i.e., a time of low estrogen and progesterone) is characterized by increased cardiac vagal activity while the luteal phase (i.e., when estrogen and progesterone peak) is characterized by an increase in sympathetic activity. Moreover, the use of oral contraceptives to maintain constant estrogen and progesterone negate cardiac vagal differences observed throughout the menstrual cycle (Teixeira et al., 2015). The male androgens (in particular testosterone) might also account for the observed sex differences in cardiac vagal activity. Animal models have reported that the administration of androgen reduces cardiac vagal activity and this effect is reversed with the administration of an androgen antagonist (Marques Neto et al., 2013). Further, humans using androgenic-anabolic steroids have shown lower vagally-mediated HRV following exercise (Maior et al., 2013). Pubertal maturation and growth in boys that is associated with rising levels of androgens may shift the relative dominance of vagal activity to decreased vagal activity compared to girls in adulthood. Such an hypothesis fits current meta-theory on the association of sex-hormones, pubertal development and the development of sex-specific psychopathology (Martel, 2013). However, given that the sample of studies included in the meta-analysis comprised pre- and post-pubertal subjects, we were not able to address this question in greater detail. Future longitudinal studies are necessary to investigate the impact of pubertal maturation, associated changes in sex-hormones, and vagal activity.

#### Differences in physical activity

Greater physical activity in boys might underlie the effects observed in our present analysis. Boys are physically more active than girls (Trost et al., 2002; Sherar et al., 2007) and greater physical activity is associated with greater vagal activity (Goldsmith et al., 1997) while poor physical health in general (Kemp and Quintana, 2013) and less physical activity (i.e., Teisala et al., 2014) are associated with reduced vagal activity. Further, physical activity can increase vagally-mediated HRV (Rennie et al., 2003), in particular in preadolescents (Buchheit et al., 2007).

Most of the included studies on resting state HRV assessed physical activity/fitness. Participants in the study by Brunetto et al. (2005) completed a health questionnaire including questions on physical activity. Further, the authors determined oxygen consumption (VO^2^) peak in an incremental exercise test and compared groups with low, mid, and high aerobic fitness. There were no group differences on RMSSD, or HF-HRV and the interaction of sex and aerobic fitness was not reported. Grandjean et al. (2004) used parent reports on physical fitness (much, average, or none) as a covariate in their analysis. The effect of physical activity on sex-differences in HRV was not reported. Gutin et al. (2005) measured physical activity with accelerometry and reported a positive association between physical activity and RMSSD. In their study boys reported significantly greater physical activity. Hedelin et al. (2000) used an incremental treadmill test with continuous analyses of VO^2^ to determine physical fitness. Exact statistics were not reported, but descriptive data points to greater aerobic capacities in boys in this sample. Michels et al. used an accelerometer to monitor physical activity and used a Eurofit fitness test battery to determine maximal oxygen uptake. Boys were more active and physical fitness was associated with HRV in boys but not in girls. Moodithaya and Avadhany (2012) estimated the daily physical activity level based on a validated physical activity questionnaire, however physical activity did not differ between groups based on age and sex. Reed et al. (2006) controlled for aerobic fitness determined by a progressive shuttle run—but there were no sex differences. Thus, sex differences reported for short-term measures of HRV might relate to differences in physical activity/fitness reported in some studies.

#### Clinical implications

We can only speculate if females gain or if males lose vagal activity during adolescence. However, it seems evident that while girls have lower vagal activity, women show greater vagal activity compared to their male counterparts and independent of age show greater mean HR. With respect to the development of psychopathology, in particular during the sensitive period of adolescence, this result has important implications. Vagal activity during a resting state is a potential biomarker of clinical relevance with respect to diagnosis, monitoring, and treatment of psychopathology. For example, reduced resting state HF-HRV is both a correlate of depression among adults (e.g., Kemp et al., 2010), and a marker of treatment response (Chambers and Allen, 2002; Chien et al., 2015) such that decreases in depressive symptoms are associated with increases in HF-HRV. Furthermore, stimulation of the vagus nerve, originally developed for the treatment of epilepsy, is a promising approach for addressing treatment refractory depression among adults (Groves and Brown, 2005; O'Reardon et al., 2006) and may have potential applicability in younger populations. Furthermore, findings from the present review and meta-analysis have implications for somatic ill-health and CVD in particular. Longitudinal studies are warranted, addressing the predictive value of sex-differences in vagal activity in children and adolescents in the development of other somatic disorders, including CVD in adulthood.

## Conclusion

Drawing on data from about 5,000 children and adolescents, we provide evidence that healthy young girls display lower resting-state vagally-mediated HRV compared to boys. These sex differences in cardiac autonomic function may represent a potential pathophysiological mechanism contributing to sex-differences in psychopathology. Longitudinal studies across pubertal development are needed to support this claim by empirical evidence.

## Author contributions

JK and JR conducted the literature research and analysis. JK and JR wrote the first draft of the manuscript. TC, JT, and MK provided important intellectual content in revising the manuscript. All authors provided final approval before submission.

### Conflict of interest statement

The authors declare that the research was conducted in the absence of any commercial or financial relationships that could be construed as a potential conflict of interest.
